# Asbestos Consumption in Mongolia: 1996–2014

**DOI:** 10.3390/ijerph15010136

**Published:** 2018-01-15

**Authors:** Naransukh Damiran, Arthur Frank

**Affiliations:** 1 Department of Environmental Health, School of Public Health, Mongolian National University of Medical Sciences, Ulaanbaatar 14210, Mongolia; 2 Dornsife School of Public Health, Drexel University, Philadelphia, PA 19104, USA; alf26@drexel.edu

**Keywords:** asbestos use, chrysotile, Mongolia

## Abstract

Asbestos is still used in Mongolia in the energy and construction sectors, among others. However, limited data is available on asbestos consumption and asbestos-related disease in Mongolia. The purpose of this paper is to present the available information on the importation of asbestos into Mongolia. We used data on annual asbestos imports between 1996 and 2014 from Mongolian Customs Statistics and the National Council on Toxic and Hazardous Substances Affairs. The uses of this material are also presented with respect to chrysotile alone. Most asbestos is used for construction. Mongolia started using asbestos in the energy and construction industries as thermal insulation in 1961. Asbestos is still allowed for use in Mongolia under the Law on Toxic and Hazards Substances. There are no asbestos mines in Mongolia, and the manufacture of asbestos-containing materials does not take place there. Thus, asbestos is mainly imported from China and Russia. Mongolia used 44,422 metric tons of asbestos-containing materials between 1996 and 2014. In Mongolia, with the current use of asbestos, there will be a continuing risk of developing asbestos-related diseases from past use, and proper oversight of asbestos-involving activities and the safe removal and disposal of asbestos must be considered.

## 1. Introduction

The World Health Organization (WHO) and The International Labor Organization (ILO) recommend that countries reduce consumption of asbestos, eventually stopping all use in order to prevent asbestos-related diseases (ARDs) such as asbestosis, as well as mesothelioma, lung cancer, and other cancers [1,2,3]. In 2015, The International Ban Asbestos Secretariat reported that all forms of asbestos are banned in more than 50 countries [4,5]. World asbestos consumption was 2.02 million metric tons in 2014, and Russia, China, and Brazil produced 88% (1.7 million metric tons) of all global asbestos [6]. While most developed countries have banned asbestos, many Asian countries are still using it [4,6,7]. Asian countries used 63.7% of the world’s asbestos between 2001 and 2007, with China and India accounting for over 50% at this time. Kazakhstan, Kyrgyzstan, the United Arab Emirates Thailand, and Uzbekistan were the top five asbestos users in Asia in the period between 2001 and 2007 [7].

Many reports document that asbestos is a human carcinogen [8,9,10]. Asbestos (in all its forms) can cause asbestosis as well as malignant mesothelioma, lung cancer, and other cancers [10]. WHO reports that 125 million people in the world are exposed to asbestos in the workplace and 107,000 people die due to ARDs every year [2]. Researchers showed relationships between ARDs and historical asbestos consumption in the UK, Hong Kong, Japan, and Germany as well as other countries [11,12,13,14]. Deaths due to mesothelioma increase by 2.4 times (95% CI 2.0–2.9) per kilogram of asbestos consumption per capita per year [15]. 

As in many other Asian countries, asbestos is still used in Mongolia [4]. The primary uses of asbestos in Mongolia are in thermal power plants, construction, and in railway companies. There is, however, limited data on both asbestos consumption and ARDs in Mongolia [4,16,17,18]. The National Health Department registered 54 cases of mesothelioma between 1996 and 2013 [19]. The quality of pathologic review remains questionable. Asbestos-related disease has not been registered in Mongolia and none of these cases were registered as asbestos-related diseases. However, if this data is correct, it shows that the average of age-specific incidence rate of mesothelioma was 19.2 per 100,000 persons, which is much higher than the world average (0.49) [19,20]. We recently reported a case of mesothelioma in Mongolia. A 47-year-old woman was diagnosed with pleural mesothelioma after 27 years of exposure to chrysotile asbestos in thermal power plants [21]. A companion article documented occupational exposure to airborne asbestos among workers of coal burning thermal power plants in Mongolia [16]. The average exposure level to airborne asbestos in thermal power plants was 9.3 times higher than the Mongolian occupational exposure limit (0.1 fiber per cubic centimeter) [16]. No articles have reported the use of asbestos in Mongolia. This paper reports the use of asbestos by product type and industrial setting in Mongolia. 

The Law on Toxic and Hazardous Substances (LTHS) has been regulating registration and controlled use of hazardous substances, including asbestos, in Mongolia since 2006. The Ministry of Environment is the main governmental body responsible for the implementation of this law and the amendment of the LTHS in 2008, when The National Council on Toxic and Hazardous Substances Affairs (NCTHSA) was established. The NCTHSA licenses companies for use or sale or manufacture or disposal of hazardous substances. Other governmental agencies overseeing customs, trade, inspection, and health are involved in the implementation and enforcement of legal regulations on hazardous chemicals within their areas. Under Article 6.1.6 of the LTHS in 2007, the government approved lists of toxic and hazardous chemicals which are restricted and prohibited for use in Mongolia. However, these lists did not include any form of asbestos. In 2010, the Ministry of Environment proposed adding asbestos to the list of toxic and hazardous chemicals prohibited for use, as it causes cancer. The government approved the proposed list with Resolution 192, and the use of asbestos was legally banned in 2010. However, there was no nationwide action plan on the implementation of the government’s decision on banning asbestos and use of substitute materials in industrial sectors. After approval of Resolution 192, the government received pressure from industries regarding the banning of asbestos, especially from thermal power plants. The power plants complained that substitute materials were expensive and not compatible for use as thermal insulation on hot surfaces. In addition, owners of thermal power plants and decision-makers in Mongolia did not fully understand the health hazards and future economic burden from asbestos use. This is the main difficulty of a total ban of asbestos in Mongolia. Due to pressure from the energy sector and inadequate awareness of asbestos hazards, the government changed the legal status on asbestos in 2011 through Resolution 176. Article of 3.1.4 of the LTHC states restrictions on the use of toxic and hazardous chemicals, which are allowed only at permitted places under strict controls for approved purposes and with restricted quantities. All forms of asbestos and asbestos-containing materials (ACMs) are allowed for use only in thermal power plants for thermal insulation and heat-resistant materials, and only with licensed quantities. Other industries are not allowed to use any form of asbestos. Therefore, the total ban of asbestos was not successful, and it is still legally allowed for some purposes [17,22,23]. 

As a requirement of the LTHS and the Law on Occupational Safety and Hygiene, thermal power plants which use asbestos have to implement precautionary and protective measures, including labeling, risk assessment, hazard communication, administrative and engineering controls, environmental monitoring, medical check-ups, and training. The Governmental Agency on Specialized Inspection monitors the use of restricted toxic and hazardous chemicals including asbestos, and enforces legal regulations. 

## 2. Materials and Methods

Mongolia does not mine asbestos, nor does it manufacture any asbestos-containing products, and all consumed asbestos is imported. We used data on annual asbestos imports as the local consumption. A pilot laboratory study in 2013 analyzed asbestos-containing materials in Mongolia, and only chrysotile was identified [24]. Two sources of information on asbestos import were used in this study. We extracted data on annual asbestos imports between 1996 and 2014 from the Registration Database of National Customs Administration of Mongolia. Data on asbestos importation before 1996 does not exist. Additionally, we obtained the amount of licensed asbestos importation between 2011 and 2014 from the NCTHSA. Under the LTHS, trading companies or users need to have a license to import and use all types of asbestos and asbestos-containing products from the NCTHSA, as of 2011 [22]. National population data was obtained from Mongolian National Statistics [25]. 

We used annual consumption of total asbestos, and use in kilograms per capita per year to calculate descriptive statistics. The annual consumption of total asbestos was converted to kilograms per capita per year using national population data. 

## 3. Results

**Figure 1:**
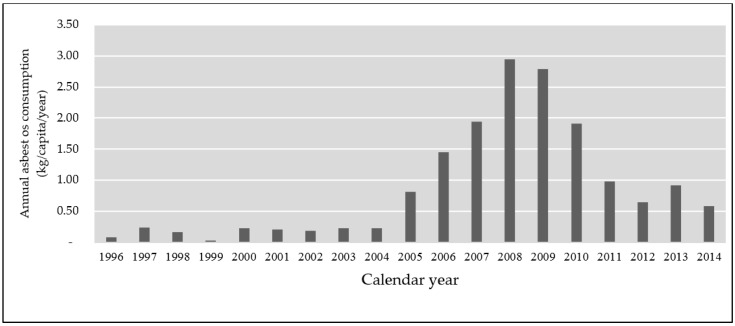
Annual per capita asbestos use (kg/capita/year) in Mongolia between 1996 and 2014.

Mongolia started using asbestos in the energy and construction industries as thermal insulation in 1961 [16]. However, there is no data on asbestos consumption in Mongolia up until 1996 due to lack of registration of asbestos and ACMs. Mongolia used 44,422 metric tons of ACMs between 1996 and 2014. The average annual consumption was 423 metric tons between 1996 and 2004. Annual asbestos consumption dramatically increased from 2005 until 2008. The consumption in 2008 was 7852 tons, the year of peak consumption. The approval of Resolution 192, which prohibits the use of asbestos reduced its consumption. Asbestos consumption decreased to 2742 tons in 2011. However, the government changed the status of the regulation on asbestos control through Resolution 176 in 2011, and asbestos use in thermal power plants was again permitted [22]. The average of annual asbestos consumption between 2011 and 2014 (2257 tons) was still higher than the average annual consumption between 1996 and 2004 (423 tons) (Figure 1).

Asbestos, in the form of chrysotile, is primary imported from China and Russia. According to Custom Statistics, 86.8%, 10.6%, and 2.5% of consumed ACMs were imported from China, Russia, and other countries, respectively. The quantity of imported ACMs from Russia decreased, while quantity of imported ACMs from other countries between 1996 and 2014 rose. The quantity of imported ACMs from China increased between 1998 and 2009, and then started to decrease after 2010 (Table 1). 

**Figure 2:**
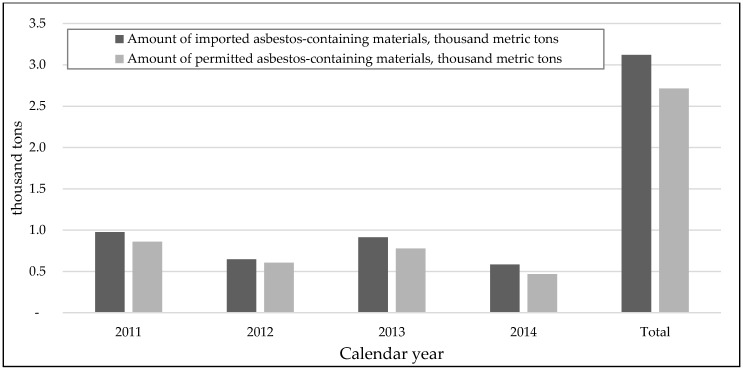
Comparisons of amount asbestos-containing materials permitted to be imported and actual importation.

After the approval of Governmental Resolution 176, the NCTHSA started to issue licenses for the import and trading activities of ACMs. The NCTHSA gave permission to such entities to import a total of 7.8 thousand tons of ACMs between 2011 and 2014. However, the total quantity of imported ACMs exceeded the permitted level (Figure 2) and 1.2 thousand tons of ACMs were illegally used in Mongolia. It appears as if some asbestos is being used for unapproved uses. In our previous study on exposure assessment to airborne asbestos at thermal power plants in Ulaanbaatar, we observed that asbestos powder was used in insulation for piping systems and for coal burning after mixing with water [16]. However, according to the data of the NTCHSA, only 60 tons of asbestos powder was imported in 2011, and as such the quantity of imported asbestos powder seems to be under-registered. 

The construction sector is still the main user of asbestos in Mongolia. According to data from the NCTHSA, 93.8% (7360 tons) of all imported ACMs between 2011 and 2014 was for construction materials. In total, 53.8% and 46.2% of the imported asbestos-containing construction materials were roof panels and wall boards, respectively. Only 0.07% of the construction materials was for noise insulation, ceiling and floor tiles, and water pipes. Others uses of ACMs included automobiles (in repair shops), heavy equipment, trucks, and locomotives and trains, and as well as in manufacturing industries (Table 2).

## 4. Discussion

Clearly, asbestos continues to be a significant worldwide health hazard, especially in Asia. This report for the first time accurately reports on the consumption of asbestos in Mongolia. Given the long lag time until disease develops, it is unfortunate that accurate data before 1996 does not exist. Prior exposures would likely be the cause of ARDs currently seen. Continued current use virtually guarantees that ARDs will persist in Mongolia well into the second half of the 21st century. Some consider consumption values of 1.0 kg/capita/year as high and over 2.0 kg/capita/year as very high with respect to commensurate documented ARDs [26]. As a rough estimation of asbestos content in imported ACMs based on the available literature, asbestos use in Mongolia averaged 0.3 kg/capita/year between 2011 and 2014 [17,27,28,29]. However, this data maybe an underestimate due to inaccurate registration of the use of asbestos in Mongolia. We believe that amount of asbestos used in Mongolia should be considered as high-level, and ARDs are likely to occur in the future. Not only does little prior data exist with regard to asbestos use, but there is no accurate, centralized data collection with respect to ARD in Mongolia. We suggest the development of an ARD registry to fully assess such diseases going forward. 

There continues to be recognition that the so-called “safe” use of asbestos does not really exist, and therefore the WHO plan to reduce and over time eliminate the use of asbestos clearly makes sense in Mongolia, as it does elsewhere. Going forward, coupling the use of chrysotile asbestos and the recording of disease in such a registry as noted above will allow for better understanding of the role of chrysotile in the development of ARDs. 

Mongolia, like many other countries in Asia, continues to use asbestos. Over 90% of this use is for construction. The increased amount of asbestos consumption between 2005 and 2008 may be related to a rise in the construction sector. There was increased construction until 2008, which then decreased due to the economic situation in Mongolia [30,31]. It is in this sector that many safer alternative materials are available and should be considered for use. Another issue in Mongolia, not captured by the current data assessment, is the significant past use of asbestos, going back decades. With current renovation and demolition, there will be a continuing risk of the development of ARDs among workers in the energy and construction sectors from past use as well, and proper oversight of such activities and the safe removal and disposal of asbestos must be considered. 

## 5. Limitation

The study has several limitations. The greatest limitation was due to the complete lack of regulations in the past with respect to the classification of asbestos as a toxic chemical substance in Mongolia. The National Customs Administration of Mongolia Office has no available registry on the amount of asbestos imported into the country before 1996. Thus, we have used data on asbestos use in Mongolia between 1996 and 2014. The amount of asbestos and ACMs imported to the country may be underestimated due to the technical capacity for identifying asbestos on the part of the National Customs Administration of Mongolia. This study only provides information on imported asbestos registration at the National Customs Administration of Mongolia and data from licensed entities for the importation and use of ACMs. Therefore, it does not provide detailed information on the use of asbestos in industrial settings and in the general environment, mainly due to a lack of registration of asbestos users and the absence of effective control systems for asbestos use in Mongolia. We could not find accurate information on the asbestos content in imported ACMs. Therefore, we roughly calculated asbestos consumption per capita based on the amount of all imported ACMs, instead of using accurate calculations of asbestos content. This is one of the limitations of the study. There is clearly continued unauthorized use of asbestos in Mongolia, giving rise to differences between data on importation and actual use. 

## 6. Conclusions

In Mongolia, with the current use of asbestos, there will be a continuing risk of developing asbestos-related diseases from past use, and proper oversight of asbestos-involving activities and the safe removal and disposal of asbestos must be considered.

## Figures and Tables

**Table 1:** Amount of imported asbestos-containing materials in Mongolia between 1996 and 2014.

Calendar Year	Origin of Asbestos-Containing Materials						Total Amount, Metric Tons
	China		Russia			Other Countries	
	Amount, Metric Tons	Percentage	Amount, Metric Tons	Percent	Amount, Metric Tons	Percentage	
1996	-	0.0%	181.6	100.0%	-	-	181.6
1997	-	0.0%	540.9	100.0%	-	-	540.9
1998	15.2	4.0%	361.1	96.0%	-	-	376.3
1999	25.2	37.0%	42.9	63.0%	-	-	68.1
2000	114.3	20.7%	437.9	79.3%	-	-	552.2
2001	15.8	3.1%	492.4	96.9%	-	-	508.2
2002	47.0	10.3%	410.7	89.7%	-	-	457.7
2003	311.8	55.5%	249.8	44.5%	0.001	<0.1%	561.6
2004	311.8	55.5%	249.8	44.5%	0.001	<0.1%	561.6
2005	1611.2	78.2%	450.3	21.8%	-	<0.1%	2061.5
2006	3323.0	88.6%	427.2	11.4%	0.48	<0.1%	3750.6
2007	4962.9	97.4%	130.6	2.6%	1	<0.1%	5094.5
2008	7726.1	98.4%	123.2	1.6%	2.5	<0.1%	7851.9
2009	7554.7	99.8%	12.3	0.2%	0	<0.1%	7567.1
2010	5099.9	96.9%	160.6	3.1%	3.5	0.1%	5260.4
2011	2241.7	81.7%	387.4	14.1%	113.5	4.1%	2742.7
2012	1571.9	84.7%	29.5	1.6%	254.9	13.7%	1856.2
2013	2345.0	87.5%	18.8	0.7%	316.2	11.8%	2680.0
2014	1297.6	74.2%	21.0	1.2%	430.2	24.6%	1748.8
Total	38,575.1	86.8%	4727.9	10.6%	1122.3	2.5%	44,421.9

Note: “-”indicates that no data was reported.

**Table 2:** Consumption of asbestos-containing materials in Mongolia between 2011 and 2014 by type.

Type of Imported Asbestos Containing Products	2011	2012	2013	2014	Total	
	Tons				Tons	Percent
Automobile parts	31.9	39.1	21.2	23.2	115.40	1.47%
Locomotive and train parts	0	0.1	0.9	2.1	3.10	0.04%
Industrial equipment parts	86.1	340.4	83.3	0.1	509.90	6.50%
Heavy equipment and truck parts	0.6	0.01	0.4	0.35	1.36	0.02%
Piping and gaskets	3.21	132.3	-	-	135.51	1.73%
Construction materials	2238.7	1223.3	2172.1	1380.7	7014.80	89.45%
Asbestos powder	60.0	-	-	-	60.00	0.77%
Airplane parts	0.0003	0.0008	-	-	0.001	<0.00%
other products	-	2	0.04	-	2.04	0.03%
Total	2420.6	1737.2	2277.9	1406.5	7842.	100.00%

Note: Data from National Council on Toxic and Hazardous Substance Affairs. “-”indicates that no data was reported. The percentages do not add up to 100% due to rounding.
